# Applying Reinforcement Learning for Enhanced Cybersecurity against Adversarial Simulation

**DOI:** 10.3390/s23063000

**Published:** 2023-03-10

**Authors:** Sang Ho Oh, Min Ki Jeong, Hyung Chan Kim, Jongyoul Park

**Affiliations:** 1 Business Department of Convergence and Open Sharing System, Seoul National University of Science and Technology, Seoul 01811, Republic of Korea; shoh0320@seoultech.ac.kr; 2 Department of Applied Artificial Intelligence, Seoul National University of Science and Technology, Seoul 01811, Republic of Korea; 3 The Affiliated Institute of Electronics and Telecommunications Research Institute, Daejeon 34044, Republic of Korea

**Keywords:** deep reinforcement learning, cybersecurity, adversarial simulation, artificial intelligence

## Abstract

Cybersecurity is a growing concern in today’s interconnected world. Traditional cybersecurity approaches, such as signature-based detection and rule-based firewalls, are often limited in their ability to effectively respond to evolving and sophisticated cyber threats. Reinforcement learning (RL) has shown great potential in solving complex decision-making problems in various domains, including cybersecurity. However, there are significant challenges to overcome, such as the lack of sufficient training data and the difficulty of modeling complex and dynamic attack scenarios hindering researchers’ ability to address real-world challenges and advance the state of the art in RL cyber applications. In this work, we applied a deep RL (DRL) framework in adversarial cyber-attack simulation to enhance cybersecurity. Our framework uses an agent-based model to continuously learn from and adapt to the dynamic and uncertain environment of network security. The agent decides on the optimal attack actions to take based on the state of the network and the rewards it receives for its decisions. Our experiments on synthetic network security show that the DRL approach outperforms existing methods in terms of learning optimal attack actions. Our framework represents a promising step towards the development of more effective and dynamic cybersecurity solutions.

## 1. Introduction

To improve the security of networked systems, red team exercises are commonly used to assess the effectiveness of their defenses by simulating different cyber-attacks. These exercises might include adversary profiles to mimic genuine advanced persistent threats and evaluate the system’s capability to safeguard against various tactics, techniques, and procedures employed by advanced attackers [1]. However, red team exercises can be time-consuming and necessitate specialized human expertise, making them an expensive means of assessing cybersecurity.

To improve the efficiency of red teaming, tools such as emulators have emerged to automate these exercises and streamline the attack simulation process [2]. Despite the automation capabilities of these red teaming tools, human experts are still critical in the planning and decision-making stages of the exercises, such as organizing tactics, techniques, and procedures through the different stages of the attack simulation campaign. These tools, such as staging frameworks, enabling scripts, and execution payloads, are designed to support human experts and simplify the red teaming process.

Adversarial simulation has become increasingly important as cyber threats continue to evolve and become more sophisticated. Traditional security systems based on predefined rules and signatures are often insufficient to defend against advanced and adaptive threats [3]. Machine learning (ML) models, on the other hand, can provide a more flexible and adaptive solution to cybersecurity by learning from historical data and evolving over time to better detect and respond to new threats [4,5,6].

As interest in using ML for cybersecurity grows, the significance of adversarial cyber-attacks against ML-based applications has become more prevalent. Adversarial simulation for cybersecurity involves the use of ML techniques to model and simulate potential cyber-attacks on a system in order to train ML models to identify and respond to these attacks in real time. This allows organizations to better understand and prepare for potential cyber threats and improve their overall cybersecurity posture. However, traditional ML-based applications have limitations as they are typically trained on historical data and have limited generalizability [7,8,9]. The rapid progress of artificial intelligence (AI) presents the possibility of AI-assisted or self-governing AI red teaming, where AI can use its superior decision-making ability, learned through AI training, to create new attack methods against complex cybersystems that human red team experts may not have considered yet [10].

This leads to our motivation to involve teaching red agents to identify and optimize attack operations in a network using deep reinforcement learning (DRL) algorithms, which is an improved method over the traditional ML model to enhance adversarial cyber-attack simulation to find more robust solutions. Reinforcement learning (RL) is a technique that can help create autonomous agents that can make optimal sequential decisions in complex and uncertain environments. Open-source learning environments, such as OpenAIGym have increased the possibilities of RL research in different application domains [11]. In recent years, the use of reinforcement learning in adversarial simulation in cybersecurity has become more popular [12,13,14,15,16]. With cyber-attacks becoming more sophisticated and the challenge of designing effective defenses against them, researchers have turned to ML techniques such as RL to develop more resilient and adaptable security systems. RL algorithms can learn optimal strategies for defending against attacks, adapting to changing threats, and evolving attack techniques. By repeatedly playing a game of offense and defense, an RL agent can learn to anticipate and defend against various types of attacks, including zero-day exploits [17]. This approach has been shown to be effective in detecting and mitigating cyber-attacks in a variety of settings, including web applications, network intrusion detection, and malware analysis.

The RL has the potential to enhance cybersecurity by enabling adaptive and automated defense systems that can learn from experience and respond to changing cyber threats in real time [18]. However, there are still significant challenges that need to be addressed to effectively apply RL in the context of cybersecurity. One of the primary challenges is the lack of training data [5,19]. Adversarial cyber-attack scenarios are often rare and complex, making it difficult to collect sufficient data to train RL models effectively. This can result in models that are underfit, meaning they do not capture the full complexity of the real-world scenarios they are designed to address. Another challenge is the difficulty of modeling complex and dynamic attack scenarios [20,21]. Cyber-attacks can be highly dynamic and adaptive, making it challenging to develop accurate models that can effectively capture the full range of potential attack strategies and tactics. This can lead to models that are overfit, meaning they are too narrowly focused on specific attack scenarios and may not generalize well to new or unexpected attack scenarios. In addition to these challenges, there is also a shortage of open-source cybersecurity-based RL experimentation environments that can help researchers address real-world challenges and improve the state of the art in RL cyber applications [22]. Without access to realistic and scalable experimentation environments, researchers may struggle to develop and test new RL-based approaches to cybersecurity.

Despite these challenges, there are efforts underway to address these issues and advance the use of RL in cybersecurity. Elderman et al. focus on cyber-security simulations in networks modeled as a Markov game with incomplete information and stochastic elements [23]. They showed the resulting game that is an adversarial sequential decision-making problem played with two agents, the attacker and the defender. Additionally, Applebaum et al. provide an analysis of autonomous agents trained with RL through a series of experiments examining a range of network scenarios [24]. Additionally, Microsoft released CyberBattleSim, which uses a Python-based OpenAIGym interface to create an initial, abstract simulation-based experimentation research platform for training automated agents using RL [25]. This platform can provide a baseline for researchers to conduct experiments, test different approaches, and develop new models that can be applied to real-world cybersecurity challenges. As technology advances, we can expect to see more RL experimentation environments that can help enhance the state of the art in RL cyber applications.

In this research, we aim to demonstrate the effectiveness of applying DRL to adversarial simulation in cybersecurity by performing the red teaming simulation that shows significant learning curves of the agent. We use simulations to model cyber-attacks and evaluate the performance. The results showed that the DRL policy was able to learn and execute effective strategies for successfully infiltrating a simulated cyber environment, highlighting the potential for DRL algorithms to be used for both defensive and offensive purposes in the field of cybersecurity.

The research involved the application of DRL to adversarial cyber-attack simulation. The use of DRL in this context allows for the creation of adaptive and automated defense systems that can learn from experience and respond to changing cyber threats in real time. One of the key contributions of this research is the significant results achieved by DRL. By modeling and simulating potential cyber-attacks and their effects on a system, the DRL-based defense systems were able to identify and respond to attacks in real time, enhancing the overall cybersecurity posture of the system. In addition to applying DRL to adversarial cyber-attack simulation, the researchers also experimented with various parameters in DRL, such as values of epsilon and epsilon decay. By varying these parameters and performing several simulations on each parameter value, the researchers were able to identify appropriate values of epsilon and epsilon decay that led to robust convergence. This finding is significant as it allows for the development of more effective and efficient DRL-based defense systems, with parameters that can be fine-tuned to specific cyber threat scenarios. Overall, the research demonstrates the potential of DRL-based approaches to enhance cybersecurity and highlights the importance of parameter tuning in achieving robust convergence and optimal performance of DRL-based defense systems in the context of adversarial cyber-attack simulation.

We organize our paper as follows. We cover the materials and methods, which explain the DRL algorithm, deep Q-learning, and simulation settings in Section 2. Section 3 presents the research results, which show the reward obtained by agents for each step. In Section 4, we discuss the results, then present conclusions in Section 5.

## 2. Materials and Methods

RL methods are able to discover approximate solutions for Markov decision processes (MDPs) by teaching policies that target the maximization of the expected reward over a specific time horizon [26]. Similar to MDPs, RL makes use of the state, action, and reward components, but instead of exhaustively searching the state space for the optimal policy, an agent interacts with the environment and chooses actions based on its current state and past rewards or penalties.

This study employs a simulated virtual environment for the agent to interact with, rather than a real network, resulting in quicker policy learning. Nonetheless, using a simulation necessitates certain simplifications that should be taken into account when implementing the learned policy on a real network.

### 2.1. Environment Settings for Simulation

The purpose of the experiment was to verify the applicability of reinforcement learning algorithms for simulating adversarial attacks in cybersecurity. We utilized the CyberBattleSim library to implement the nodes in the virtual environment to simulate attack scenarios, then apply a reinforcement learning algorithm to derive the results.

To achieve this, we set our environment as a company with computers to attack, then the following steps were taken:

1. Investigation of vulnerabilities that may exist in virtual company computers.
2. Configuration of nodes based on the findings from the investigation.
3. Creation of a virtual environment to simulate the acquisition of confidential documents hidden within the company’s computers.
4. Implementation of reinforcement learning to determine the effectiveness of the simulated environment.

### 2.2. Deep Q-Learning (DQL)

In cases where the transition function or probability distribution of a state variable is known, Q-learning is capable of determining the best course of action. Q-learning is based on the estimation of a value function, which is a set of Q-values. The Q-learning method [27] calculates Q-values for each state–action combination $\left(s_{t},a_{t}\right)$. Once the final Q-values have been determined, the state of the environment can be used to select the optimal action. At the start of the process, Q-values are initialized to an arbitrary real number. In iteration t, the agent evaluates the reward in each $\left(s_{t},a_{t}\right)$ combination. The algorithm updates the Q-values iteratively based on the immediate reward $r_{t}$ and the Q-values of the next state–action combination $Q\left(s_{t+1},a_{t+1}\right)$, as shown in Equation (1). The discount factor, γ, which has a range of 0 to 1, regulates the effect of future rewards on current rewards.

(1) $$Q\left(s_{t},a_{t}\right)\leftarrow r_{t}+\gamma \underset{a_{t+1}}{\max}\left\{Q\left(s_{t+1},a_{t+1}\right)\right\}$$

Regardless of the policy being followed, the Q-values are adjusted so that they eventually reach an optimal action-value function Q* [28]. One of the interesting features of Q-learning is that it can produce state–action pairs using various sampling techniques. A common sampling method is the ε-greedy action selection, as shown in Equation (2), where ε is a value in the range (0, 1].

(2) $$a_{t}=\{\begin{array}{c} arg\underset{a\in A}{\max}Q\left(s_{t},a\right)\\ a\;\sim \;A \end{array}\;\begin{array}{c} with\;probability\;1-\varepsilon ,\\ otherwise. \end{array}$$

The function approximator, such as a deep neural network, can be utilized to estimate the Q-values, instead of using a Q-table [29,30]. The parameter set for the approximator is denoted as θ and the network is represented as Q(s,a;θ). The network is then optimized to estimate the Q-values, as defined in Equation (1) [30,31]. In DQL, there are two networks involved, the Q-network (Q(s,a;θ)) and a target Q-network ($\hat{Q}\left(s,a;\theta^{-}\right)$*)* [29,32]. The Q-network is used to determine the optimal action and the target Q-network is used to generate the target value for updating the parameters (θ) of the Q-network. The Q-network is updated at each iteration by minimizing the mean-square error between the target Q-network ($\hat{Q}\left(s^{\prime },a^{\prime };\theta^{-}\right)$) and the current Q-network (Q(s,a;θ)) using a loss function, as described in Equation (3).

(3) $$L_{i}\left(\theta_{i}\right)=E\left[{\left(r+\gamma arg\underset{a^{\prime }}{\max}\hat{Q}\left(s^{\prime },a^{\prime };\theta^{-}\right)-Q\left(s,a;\theta_{i}\right)\right)}^{2}\right]$$

The exploration and exploitation trade-off must be carefully considered in a DQL as exploration evaluates potential actions while exploitation utilizes previous experience. The DQL also utilizes replay memory, which is a database storing the agent’s experiences [32]. To update the Q-networks, experiences are randomly selected from the replay memory [33].

#### 2.2.1. States

In our research, the state is composed of various values such as the node’s name, information, and vulnerability. These values provide the agent with a complete understanding of the current state of the environment. Additionally, there is a reward associated with occupying the node, which serves as a motivation for the agent to take specific actions. The reward can be seen as a reinforcement signal that guides the agent towards optimal behavior. The state, along with the available actions and their corresponding rewards, forms the basis for decision-making in DQL.

#### 2.2.2. Actions

The attacker has three options for actions: (1) exploiting a vulnerability that is local to the system, (2) exploiting a vulnerability that is remotely accessible, or (3) connecting to another host through lateral movement. The actions require different parameters, such as specifying which vulnerability to use or which user credentials to access. There are also specific requirements that must be met before each action can be taken, such as discovering the target host or having knowledge of the necessary credentials. The consequences of each action can include discovering new hosts, obtaining sensitive information, or gaining control of another host.

#### 2.2.3. Rewards

The reward is the feedback that the environment provides in response to the agent’s actions, and it plays a crucial role in shaping the interaction between the agent and the environment. This research considers both the cost of the attacker’s actions and the impact of action utilization on the penetration testing process when designing the reward system. The cost of an action and its variation are used to calculate the environmental reward value for the agent’s current action, determining the reward or penalty associated with its use. Specifically, this study provides a positive reward when the attacker successfully acquires the node and a negative reward otherwise.

### 2.3. Simulation Procedure

The simulations in the system take place as attacker games, played over a specific network structure. Each game has a set number of turns or iterations, which end either when the attacker has successfully completed their objective or when the maximum number of turns has been reached. During each turn, the attacker takes an action and receives updated information about the environment as well as a reward based on pre-defined values linked to the action taken, the outcome, and the importance of the target host. If the attacker accomplishes their objective, they receive a significant reward and the episode comes to an end.

Here is the simulation scenario:

1. The first entry point is allowing remote connection to a conference room PC for a meeting.
2. It is assumed that the administrators have uploaded passwords and credential tokens related to sudo permissions in a GitHub private repository.
3. A GitHub token is found in the bash history of the conference room PC and can be used to access the GitHub private repository.
4. Using the obtained sudo permissions from GitHub, the attacker tries to access files.
5. Using the sudo permissions, the attacker accesses the internet browser’s cookie history and retrieves the administrator’s session ID.
6. The obtained session information is used to access the company PC as an administrator and obtain confidential information in the confidential folder.

The terminal condition is that the execution stops if the attacker obtains the confidential information or if the iteration exceeds 500 steps.

## 3. Results

In this section, we present the cumulative reward outcomes of our DQL agent in comparison to random search. The results demonstrate that utilizing DRL methods enabled the agent to acquire an efficient attack technique within a short learning period. The findings indicate that the RL policy managed to acquire and execute effective tactics for accomplishing the goal of infiltrating a simulated cyber environment, underscoring the potential of RL algorithms to be utilized for both offensive and defensive purposes in the field of cybersecurity. The graphs presented in this section will show the reward obtained by agents for each step. As the step progresses, the agent will learn in the direction of obtaining a higher reward, and the interesting part to observe here is the difference in reward between DQL and random search as the step progresses.

### 3.1. DQL vs. Random Search

**Figure 1:**
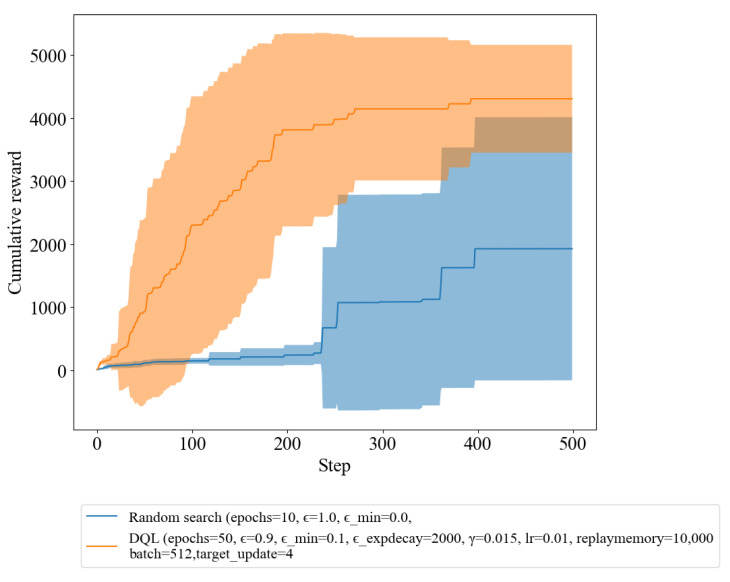
Reward of random search and DQL with fixed epsilon and epsilon Decay.

Our research aimed to evaluate the performance of two policies, the random policy and the DQL policy. In Figure 1, the results of the experiment are shown in a graph, where the blue line represents the reward obtained using the random policy, and the orange line represents the reward obtained using the DQL policy. The agent’s objective was to reach the terminal condition, a single path, while maximizing the reward within 500 iteration steps. It is clear from the graph that the DQL policy outperforms the random policy, as the orange line gradually increases as step progresses, indicating a continual improvement in the decision-making abilities of the agent.

Table 1 provides statistics of the number of iteration steps taken by each method to reach the terminal condition, which signifies convergence to the optimal solution. By analyzing the table, we can assess the speed at which each method meets the convergence condition. On average, DQL takes only 146.14 steps to converge, while random search takes an average of 425.30 steps. Moreover, DQL has demonstrated a minimum of only 24 steps to reach the convergence condition, whereas random search took 238 steps. Therefore, the table highlights the superiority of DQL over random search in terms of convergence speed, which is a critical factor in achieving efficient and effective learning in RL applications.

This result demonstrates that the DQL policy is a more effective approach compared to the random policy. The DQL policy’s ability to continually improve its decision-making over time results in a more efficient and effective agent that is able to reach the terminal condition while maximizing the reward.

**Figure 2:**
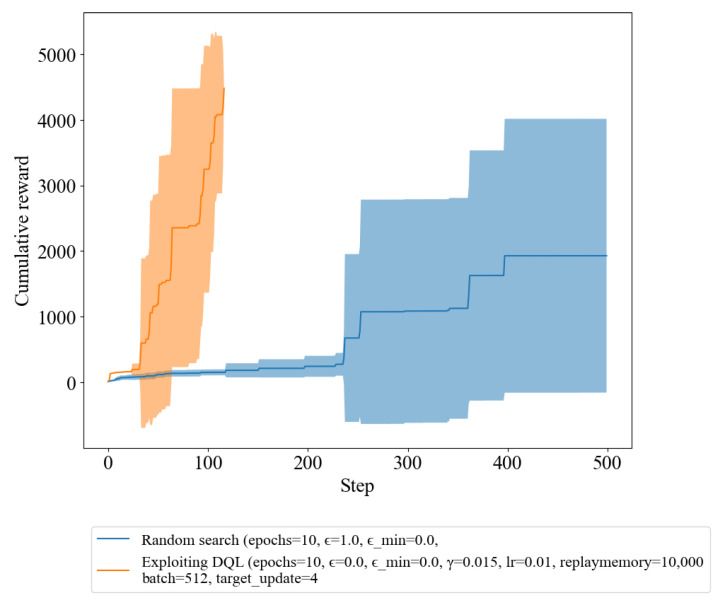
Reward of random search and exploiting DQL.

In Figure 1, we demonstrate that the DQL policy outperformed compared to random policy in terms of cumulative reward by reaching the terminal condition. In Figure 2, we experiment with the exploiting (pre-trained) DQL compared with random search. The terminal condition of the experiment was to reach a single path and achieve the highest possible reward within 500 iteration steps. As we can observe in Figure 2, the agent trained with the exploiting DQL policy was able to reach this terminal condition within the allotted steps and achieve a higher reward compared to the random policy. In contrast, the agent using the random policy was not able to satisfy the terminal condition and achieved a lower reward. This supports the conclusion that the agent trained with the DQL policy was able to effectively learn and improve its decision-making capabilities.

**Figure 3:**
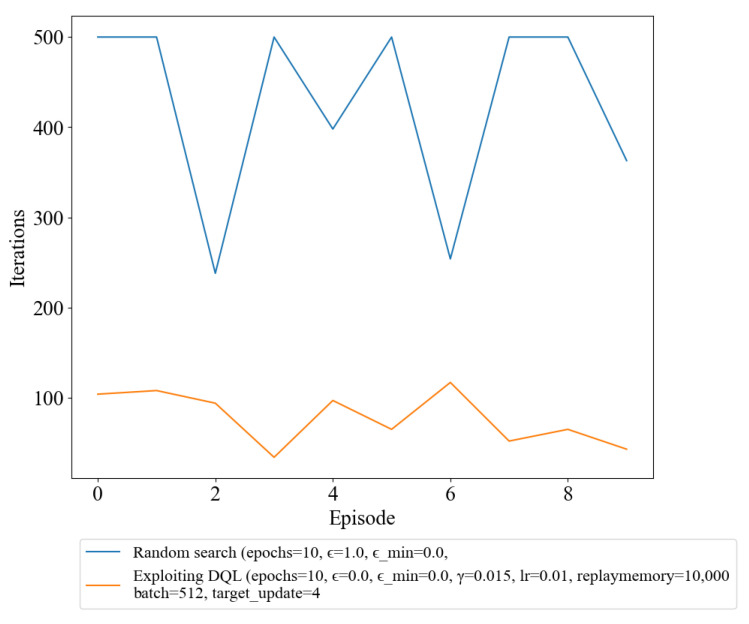
Number of Iterations per episode for random search and DQL.

Additionally, we found that the iteration step comes to an end faster in the exploiting DQL policy compared to the random search policy, as shown in Figure 3. The results of the iteration step in a shorter period of time in the DQL policy indicate that it has the ability to identify and reach the terminal condition more efficiently. The ability to reach the terminal condition faster in the DQL policy is an important factor to consider, as it provides evidence that the training process has effectively improved the policy’s decision-making capabilities. In other words, the policy has learned to make the right decisions more quickly and with greater accuracy, resulting in a faster resolution of the episode.

Table 2 presents statistical information on the number of iteration steps taken by each method to reach the terminal condition, indicating convergence to the optimal solution. The results show that exploiting DQL takes an average of only 77.90 steps to converge, which is significantly faster than normal DQL, while random search takes an average of 425.30 steps. Additionally, DQL has demonstrated a minimum of 34 steps to reach the convergence condition, whereas random search took 238 steps. Thus, Table 2 emphasizes the outstanding performance of exploiting DQL over random search in terms of convergence speed. These results highlight the importance of exploring novel techniques, such as exploiting DQL, to enhance the performance of RL algorithms.

Furthermore, this ability to reach the terminal condition more efficiently also suggests that the policy is more capable of navigating the environment in a more optimized way, reducing the number of iteration steps required to complete an episode. This not only leads to a faster resolution of the episode but also results in a more efficient use of resources and a reduction in computational costs.

In conclusion, the results of the experiment provide valuable insights into the effectiveness of the training process, demonstrating the improvement in decision-making capabilities and efficiency in reaching the terminal condition in the trained policy. These findings highlight the importance of using training methods to improve the performance of an agent in an RL environment.

### 3.2. Comparison of Learning Rates Based on Epsilon

In this experiment, we investigated the effect of epsilon in DQL on the learning rate of the agent. By comparing the learning rate for different epsilon values, we were able to observe how the exploration–exploitation trade-off impacted the overall performance of the agent. The results of this comparison provide insights into how the choice of epsilon value can impact the learning process and inform future design decisions.

**Figure 4:**
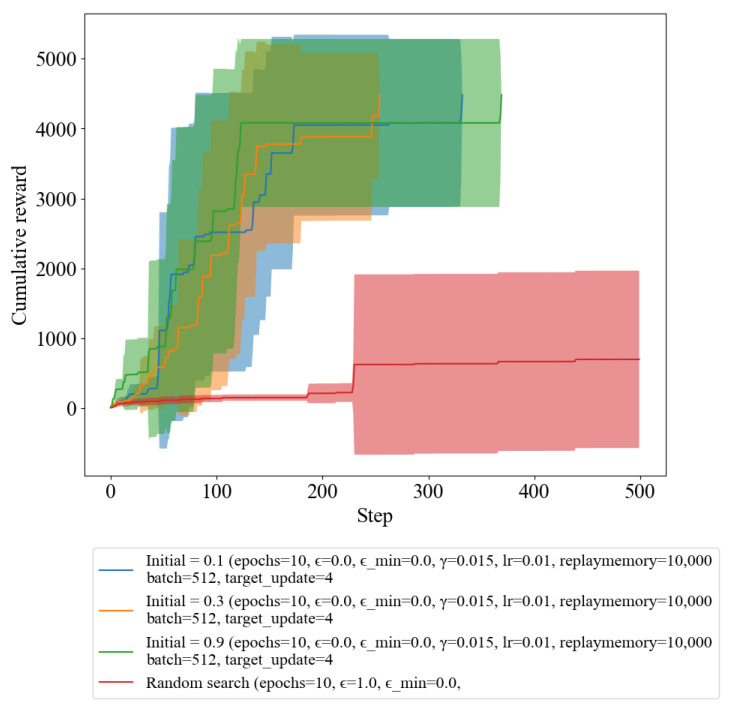
Reward of random search and DQL with varying epsilon values.

In Figure 4, we compared the results of cumulative rewards by varying the initial value of epsilon. The values used were 0.1, 0.3, and 0.9, which are blue, orange, and green, respectively. The objective of the agent was to reach the terminal condition while maximizing the reward within 500 iteration steps. The comparison of the results based on the epsilon value was conducted to determine the impact on the agent’s learning rate and decision-making abilities. The initial value of epsilon determines the exploration–exploitation trade-off in the reinforcement learning process. A higher initial value of epsilon would result in more exploration and less exploitation, while a lower initial value of epsilon would result in more exploitation and less exploration. The results show that a suitable initial value of epsilon is essential for fast convergence of the agent towards an optimal policy.

In Figure 4, the green graph indicates that the agent with a high initial value of epsilon takes longer to converge and shows a lot of fluctuations in its reward trajectory. This can be attributed to the high exploration rate, which leads to inefficient exploitation of the learned policy. On the other hand, the blue and orange graphs show faster convergence and less fluctuation, indicating that the agents with lower initial values of epsilon are better able to exploit the learned policy. Therefore, it can be concluded that selecting an appropriate initial value of epsilon is important for efficient and stable learning of a good policy. A suitable initial value of epsilon ensures that the agent converges quickly and performs optimally, without being too unstable.

Table 3 provides statistics on the number of iteration steps taken by each epsilon value and random search method to reach the terminal condition. The results reveal that the epsilon value of 0.1, 0.3, and 0.9 took an average of 123.30, 134.10, and 112.60 steps, respectively, while random search took 473.10 steps. Although DQL outperforms random search, it is crucial to select appropriate epsilon values depending on the training priority in balancing the exploration and exploitation trade-off to achieve optimal performance in RL applications. Higher epsilon values are associated with a high exploration rate, which can result in less exploitation of the learned policy. On the other hand, lower epsilon values indicate faster convergence and less fluctuation, suggesting that the agent can exploit the learned policy effectively.

### 3.3. Cumulative Reward vs. Step with Fixed Epsilon and Variable Epsilon Decay

In this section, we aimed to investigate the impact of epsilon decay on the performance of the DQL algorithm. We adjusted the degree of decay, which regulates the rate at which epsilon decreases. The results of this adjustment were recorded and analyzed to determine the optimal decay rate for the DQL algorithm.

**Figure 5:**
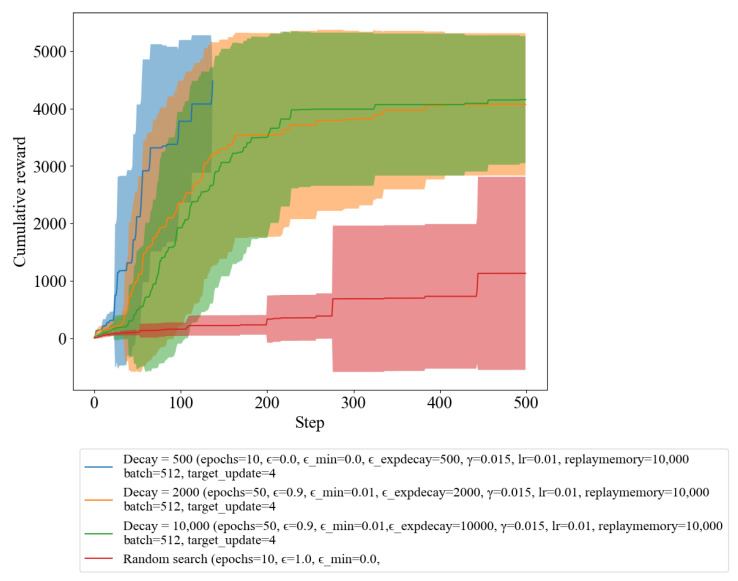
Reward of random search and DQL with varying epsilon decay.

The impact of the epsilon decay rate on the convergence rate and stability of the learned policy is analyzed in Figure 5. We experimented with epsilon decay of 500, 2000, and 10,000, which are the blue, orange, and green lines in Figure 5, respectively. The results showed that a lower decay rate resulted in a slower convergence rate and a more unstable policy, while a higher epsilon decay rate resulted in a faster convergence rate and a more stable policy. This highlights the importance of carefully choosing the epsilon decay rate to ensure that the DQL algorithm could learn the optimal policy effectively. 

**Figure 6:**
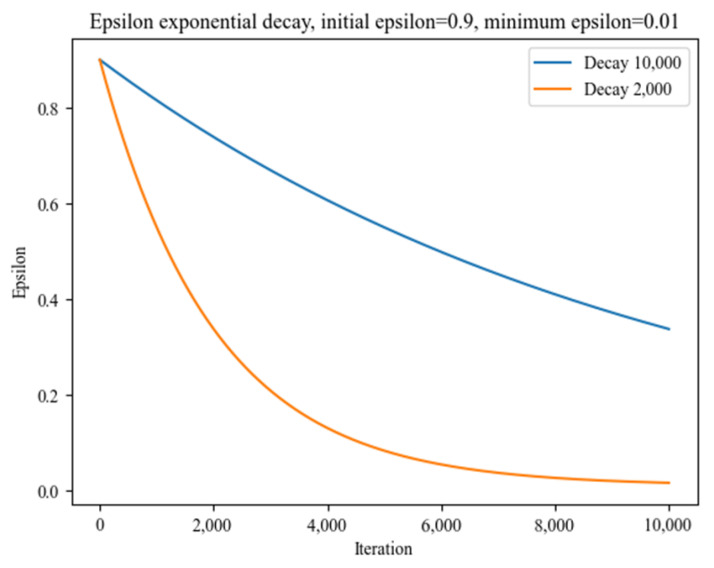
Impact of epsilon decay on the rate of epsilon values.

We investigated the impact of epsilon decay on the rate of epsilon value in Figure 6. Our findings showed that as decay increased, epsilon decreased at a slower rate. This resulted in a model that tended to exhibit greater randomness in its actions even when the episode number was sufficiently high, and the agent had learned enough. This highlights the importance of considering the decay rate when training the agent to ensure it does not exhibit random behavior at the end of the training process.

The statistics of the number of iteration steps taken by each epsilon decay to reach the terminal condition shown in Table 4. The epsilon decay of 500, 2000, and 10,000 takes an average of 67.90, 152.70, and 161.90 steps, respectively, while random search takes 472.2 steps. A lower epsilon decay resulted in a suboptimal policy, while a higher epsilon decay resulted in a poorly converged policy. Therefore, it is important to carefully choose the decay rate to ensure that the DQL algorithm is able to learn the optimal policy.

## 4. Discussion

The findings from our experiment clearly demonstrate the effectiveness of utilizing DRL methods over random search in the task of infiltrating a simulated cyber environment. As the number of iterations increased, the DQL policy displayed a remarkable improvement in decision-making, which resulted in a higher reward compared to the random policy. This can be observed from Section 3.1, where the DQL policy consistently outperformed the random policy in terms of convergence speed, with an average of 146.14 steps to reach the terminal condition compared to 425.30 steps for random search. The efficiency of the DQL policy in reaching the terminal condition is reflected in the faster resolution of the episode, more efficient use of resources, and reduction in computational costs. Exploiting DQL further improved the performance of the algorithm, with an average of only 77.90 steps to reach the terminal condition. This result highlights the potential of novel techniques, such as exploiting DQL, to enhance the performance of RL algorithms in the field of cybersecurity. 

We also examined the impact of the initial value of epsilon on the convergence and stability of the learned policy. The epsilon value plays a critical role in balancing the exploration and exploitation trade-off in RL applications. Our results indicate that an epsilon value of 0.1, 0.3, and 0.9 took an average of 123.30, 134.10, and 112.60 steps, respectively, to reach the terminal condition, while random search took 473.10 steps. These results showed that the initial value of epsilon is a critical factor that affects the convergence and stability of the policy. Our results demonstrated that a slower convergence rate led to more fluctuating behavior, while a faster convergence rate led to less fluctuating behavior.

Moreover, we also analyzed the impact of the epsilon decay rate on the performance of the DQL algorithm. Our results indicate epsilon decay of 500, 2000, and 10,000 takes an average of 67.90, 152.70, and 161.90 steps, respectively, while random search takes 472.2 steps. A lower epsilon decay resulted in a suboptimal policy, whereas a higher epsilon decay resulted in a poorly converged policy. Therefore, careful selection of the decay rate is crucial to ensure that the DQL algorithm is able to learn the optimal policy. 

Additionally, the results of our experiments have shown the impact of epsilon decay. When epsilon decay increased, epsilon decreased at a slower rate. This means that the agent has a greater likelihood of selecting random actions, even in the later stages of learning, when it should have learned the best policy. This phenomenon can be seen as the agent continues to select random actions even after enough episodes have passed for it to be considered well trained. One possible explanation for this behavior is that the agent continues to explore its environment even after it has learned a good policy. This can be seen as a benefit, as it allows the agent to refine its policy in response to changes in its environment. However, it can also result in suboptimal performance if the agent becomes too confident in its actions and fails to account for changes in its environment.

Our research highlights the significance of considering the decay rate carefully when training reinforcement learning agents. Finding the right balance between exploration and exploitation is crucial for optimal performance, which may vary based on the task and environment. Our results show the superiority of the DQL policy over the random policy in terms of reward and convergence rate, and there is room for further improvement by optimizing the parameters and hyperparameters of the DQL algorithm or exploring alternative reinforcement learning algorithms.

## 5. Conclusions

Our research findings provide clear evidence of the superiority of the DQL policy over the random policy in terms of both reward and convergence rate. The DQL policy exhibited a significant improvement in decision-making ability as the number of iterations increased, resulting in a higher reward compared to the random policy.

Our analysis of the effect of the initial value of epsilon on the convergence and stability of the learned policy revealed that the initial value of epsilon determines the speed of convergence and stability. Additionally, our results showed that epsilon decay affects the rate of decrease in epsilon, and a higher epsilon decay leads to a slower decrease in epsilon. This can cause the agent to continue making random actions even after it has been well trained. While this exploration can refine the policy, it can also result in suboptimal performance if the agent becomes too confident in its actions. Future studies should explore alternative decay strategies that balance the need for exploration with the need for exploitation.

In conclusion, achieving the right balance between exploration and exploitation is crucial for optimal performance and may vary depending on the specific task and environment. There is an opportunity for further optimization of the parameters and hyperparameters of the DQL algorithm, and exploring alternative reinforcement learning algorithms could lead to even more effective policies and provide valuable insights into the field of reinforcement learning. It is important to note that while our findings are promising, the proposed DQL algorithm has limitations and may not be applicable to all scenarios. Future research should investigate the limitations and applicability of the DQL algorithm in different contexts.

## 6. Limitations and Future Works

Our limitation of this research is that, since this research focused on the application of the RL algorithm to the cybersecurity field, it was conducted in a simulated environment that did not capture the complexity of real-world cyber-attacks, which may include multiple attackers and defenders, various attack vectors, and dynamic changes in the environment. Furthermore, the impact of varying the network architecture was not investigated in this study, and it is possible that using more complex network architectures could further improve the performance of the algorithm.

There is a lot of scope for further exploration in the field of utilizing DRL methods for cybersecurity tasks. One possible direction for future research is to incorporate more complex network architectures such as convolutional neural networks (CNNs) or recurrent neural networks (RNNs) to improve the performance of the DQL algorithm. Additionally, different learning techniques such as actor–critic or policy gradient methods could be explored to determine whether they outperform DQL in this application. Another direction is to examine the performance of the algorithm on real-world cybersecurity datasets and applications.

## Figures and Tables

**Table 1:** Iteration steps taken to convergence for each method.

Methods	Iteration Steps			
	Average	Std	Max	Min
DQL	146.14	107.88	500	24
Random search	425.30	101.26	500	238

Std—Standard deviation.

**Table 2:** Comparison of iteration steps to convergence between DQL and random search.

Policy	Iteration Steps			
	Average	Std	Max	Min
Exploiting DQL	77.90	28.09	117	34
Random search	425.30	101.26	500	238

Std—Standard deviation.

**Table 3:** Comparison of iteration steps to convergence for varying epsilon values and random search.

Epsilon	Iteration Steps			
	Average	Std	Max	Min
ϵ = 0.1	123.30	83.90	333	47
ϵ = 0.3	134.10	62.50	255	65
ϵ = 0.9	112.60	90.70	370	37
Random search	473.10	80.70	500	231

Std—Standard deviation.

**Table 4:** Comparison of iteration steps to convergence for varying epsilon decay and random search.

ϵ-Decay	Iteration Steps			
	Average	Std	Max	Min
500	67.90	35.36	138	25
2000	152.70	139.20	500	36
10,000	161.90	123.70	500	41
Random search	472.20	67.10	500	277

Std—Standard deviation.

## Data Availability

Data is not available due to privacy.
